# Effect of angiotensin-converting enzyme inhibitors and angiotensin II receptor blockers on cardiovascular events in patients with heart failure: a meta-analysis of randomized controlled trials

**DOI:** 10.1186/s12872-017-0686-z

**Published:** 2017-10-05

**Authors:** Chenhui Tai, Tianyi Gan, Liling Zou, Yuxi Sun, Yi Zhang, Wei Chen, Jue Li, Jian Zhang, Yawei Xu, Huihe Lu, Dachun Xu

**Affiliations:** 1 Department of Cardiology, The Second Affiliated Hospital of Nantong University, 6 Northern Haierxiang Road, Nantong, China; 2 Department of Cardiology, Shanghai Tenth People’s Hospital, Tongji University School of Medicine, 301 Yanchang Road, Shanghai, China; 3 State Key Laboratory of Cardiovascular Disease, Heart Failure Center Fuwai Hospital, National Center for Cardiovascular Diseases, Chinese Academy of Medical Sciences and Peking Union Medical College, Beijing, China; 4 Institute of Clinical Epidemiology and Evidence-based Medicine, Tongji University School of Medicine, 1239 Siping Road, Shanghai, China; 5 Department of Cardiology, First Hospital of Lanzhou University, Lanzhou, Gansu China

**Keywords:** Heart failure, ACEIs, ARBs, Meta-analysis, Mortality

## Abstract

**Background:**

Heart failure (HF) remains a significant cause of morbidity and mortality. Multiple trials over the past several years have examined the effects of both angiotensin-converting enzyme inhibitors (ACEIs) and angiotensin II receptor blockers (ARBs) in the treatment of left ventricular dysfunction, both acutely after myocardial infarction and in chronic heart failure. Yet, there is still confusion regarding the relative efficacy of rennin-angiotensin-aldosterone system (RAAS) inhibition. Our study was conducted to assess efficacy of ACEIs and ARBs in reducing all-cause and cardiovascular mortality in heart failure patients.

**Methods:**

We included randomized clinical trials compared ACEIs and ARBs treatment (any dose or type) with placebo treatment, no treatment, or other anti-HF drugs treatment, reporting cardiovascular or total mortality with an observation period of at least 12 months. Data sources included Pubmed, EMBASE, the Cochrane Central Register of Controlled Trials. Dichotomous outcome data from individual trials were analyzed using the risk ratio measure and its 95%CI with random-effects/ fixed-effects models. We performed meta-regression analyses to identify sources of heterogeneity. All-cause mortality and CV mortality were thought to be the main outcomes.

**Results:**

A total of 47,662 subjects were included with a mean/median follow-up ranged from 12 weeks to 4.5 years. Of all 38 studies, 32 compared ACEIs with control therapy (included 13 arms that compared ACEIs with placebo, 10 arms in which the comparator was active treatment and 9 arms that compared ACEIs with ARBs), and six studies compared ARBs with placebo. ACEIs treatment in patients with HF reduced all-cause mortality to 11% (risk ratio (RR): 0.89, 95% confidence interval (CI): 0.83–0.96, *p* = 0.001) and the corresponding value for cardiovascular mortality was 14% (RR: 0.86, 95% CI: 0.78–0.94, *p* = 0.001). However, ARBs had no beneficial effect on reducing all-cause and cardiovascular mortality. In head-to-head analysis, ACEIs was not superior to ARBs for all-cause mortality and cardiovascular deaths.

**Conclusions:**

In HF patients, ACEIs, but not ARBs reduced all-cause mortality and cardiovascular deaths. Thus, ACEIs should be considered as first-line therapy to limit excess mortality and morbidity in this population.

**Electronic supplementary material:**

The online version of this article (10.1186/s12872-017-0686-z) contains supplementary material, which is available to authorized users.

## Background

Chronic heart failure (HF) has one of the highest morbidity and mortality rates for cardiovascular diseases worldwide, which affects 1–2% of the adult population in developed countries [1]. To lower the risk of adverse clinical outcomes is therefore extremely important in the therapy of this chronic disease.

It is generally accepted that one of the pathophysiological mechanisms of heart failure is excess activation of the rennin angiotensin aldosterone system (RAAS), so that blockade of the RAAS is one of the key therapeutic targets in patients with HF [2–6]. Recent years, a lot of clinical trials have confirmed that suppression of RAAS (angiotensin-converting enzyme inhibitors (ACEIs) and angiotensin II receptor blockers (ARBs)) reduces cardiovascular (CV) events in patients with heart failure [7–13].

Moreover, the cardioprotective effects of RAAS were recently called into question. The SOLVD study [5] demonstrated that the addition of enalapril to conventional therapy significantly reduced mortality and hospitalization due to heart failure in HF patients. In the ELITE study [14], it was found that treatment with losartan was associated with lower all-cause mortality than captopril. But, in several head-to-head trials (such as the ELITE II study, the VALIANT study, the RESOLVD study and the OPTIMAAL study), ARBs did not significantly reduce cardiovascular mortality as compared with ACEIs [9–12].

Recent meta-analysis reported that in HF patients with hypertension [15] and diabetes [16], treatment with ACEIs resulted in a significant further reduction in all-cause and CV mortality, whereas ARBs had no benefit on these outcomes. These studies indicate that there are different clinical outcomes between ACEIs and ARBs among patients with heart failure.

In light of these conflicting reports, the present meta-analysis was conducted to assess the efficacy of ACEIs and ARBs on all-cause and CV mortality in patients with heart failure.

## Methods

### Literature search

We searched the database through PubMed, EMBASE, and the Cochrane Central Register of Controlled Trials for randomized clinical trials (RCTs) from November 1977 to June 2017 using Medical Subject Heading ‘antihypertensive agents’ or ‘angiotensin II type 1 receptor blockers’ or ‘angiotensin-converting enzyme inhibitors’ and ‘heart failure’. Additionally, studies in the reference lists of the identified articles were also hand searched. The search was limited to RCTs, human subjects and English. The process was strict to the Preferred Reporting Items for Systematic Reviews and Meta-Analyses (PRISRMA) statement [17].

### Study eligibility

Studies were deemed eligible if they: 1) were RCTs, targeting HF patients with reduced ejection fraction (HFrEF, left ventricular ejection fraction ≤45%), with a median or mean follow-up of more than 12 months; 2) compared ACEIs and ARBs treatment (any dose or type) with placebo treatment, no treatment, or other anti-HF drugs treatment; 3) reported cardiovascular or total mortality. When the outcomes obtained from the same population in different publications, only the latest report was included in the analysis.

### Data extraction and quality assessment

Two independent investigators (Y. X. and D. X.) extracted data from these reports, and disagreements were resolved by consensus. After excluding the unrelated studies, the following data were extracted: study characteristics (author, publication year, sample size, follow-up period), population baseline characteristics (age, sex, cause of heart failure, risk factors) and end-points. Study quality was assessed using the Jadad score, which is a five-point quality scale, with low quality studies having a score of <2 and high-quality studies a score of ≥3 [18].

### Endpoint

All-cause mortality and CV mortality were thought to be the main outcomes.

### Statistical analysis

Dichotomous outcome data from individual trials was analyzed using the risk ratio (RR) measure and its 95% confidence interval (CI) [19]. Overall effect was estimated using the Mantel-Haenszel method for RRs [20, 21].

Heterogeneity was evaluated using χ^2^ tests and I^2^ statistics. Studies were considered statistically heterogeneous if I^2^ > 50% and *p* ≤ 0.05. If heterogeneity between studies were identified, a random-effects model was applied. Otherwise, a fixed-effects model was taken instead [22]. Publication bias was assessed with funnel plots and the Begg regression test [22].

In sensitivity analysis, we removed anyone of the study at a time and repeated the meta-analysis to ensure that no single study would be responsible for the significance of any result separately [22].

Meta-regression was conducted to explore the potential heterogeneity related to the participants (age, cause of HF, left ventricular ejection fraction, and follow-up weeks), the agent used (different types). *P* < 0.05 was considered statistically significant [22].

Meta-analysis was performed by the Review Manager software (Version 5.0. Copenhagen: The Nordic Cochrane Centre, the Cochrane Collaboration) and the Stata software (version 12.0; Stata Corporation, College Station, TX).

## Results

### Eligible studies and baseline characteristics

**Fig. 1:**
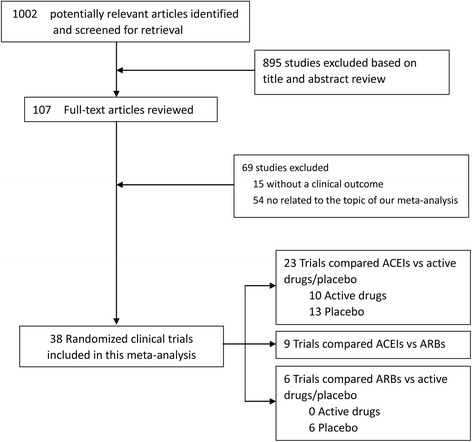
Flow diagram of selection strategy. ACEI, angiotensin-converting enzyme inhibitor, ARB, angiotensin II receptor blocker

Initial search identified 1002 reference articles, of these 107 relevant articles were selected and reviewed. Then, several studies were further excluded because they were publications from the same trial (*n* = 7) or reported of the end points other than cardiovascular events or death (*n* = 15) or used RAAS inhibitors simultaneously in both trial arms (*n* = 7) or were not relevant (*n* = 40). Finally, 38 RCTs assessing the association of cardiovascular outcome or cardiovascular or total mortality with ACEIs or ARBs were included in the meta-analysis [2–14, 22–47]. As shown in Fig. 1, literature research process was summarized by a chart flow. Baseline characteristics of all selected studies are detailed in Table 1. A total of 47,662 subjects were included with a mean/median follow-up ranged from 12 weeks to 4.5 years. Of all 38 studies, six (*n* = 8404) trials compared ARBs with placebo [13, 43–47], while 32 trials (*n* = 39,254) compared ACEIs with various control therapies (13 arms (*n* = 10,134) compared ACEIs [2–6, 23–30] with placebo treatment; 10 arms (*n* = 8714) in which the comparator was active treatment [7, 8, 31–38]; and 9 arms (*n* = 20,406) compared ACEIs with ARBs [9–12, 14, 39–42]). Two independent investigators (Y. X. and D. X.) assessed the quality of the studies included. There were 32 studies of good quality (Jadad score ≥ 3) with low risk of bias and six studies of low quality (Jadad score < 3) with high risk of bias.

**Table 1:** Study characteristics

Study, year	No of patients	Drugs		Baseline characteristics			Follow-up, w	Cause of heart failure					Risk factors			Jadad Score
		Treatment	Control	Men,%	Age, y	LVEF, %		MI, %	HTN,%	ICM, %	NICM, %	VHD, %	DM, %	HTN,%	AF,%	
ARBs vs Controls																
Havranek [43], 1999	218	Irbesartan	Placebo	82	60	≤0.40	12	–	–	67	–	–	–	–	–	2
STRETCH [45], 1999	844	Candesartan	Placebo	68	62	0.35–0.45	12	–	29	71	2	2	–	–	–	4
SPICE [44], 2000	270	Candesartan	Placebo	69	66	<0.35	12	–	4	71	16	11	48	34	24	5
ARCH-J [47], 2003	292	Candesartan	Placebo	78	64	≤0.45	24	25	7	–	57	8	–	–	–	4
Val-HeFT [46], 2001	5010	Valsartan	Placebo	80	63	≤0.40	100	–	7	57	31	–	26	–	12	5
CHARM-Alternative [13], 2003	2028	Candesartan	Placebo	68	67	≤0.40	135	–	6	68	–	–	27	50	25	5
ARBs vs ACEIs																
REPLACE [42], 2001	378	Telmisartan	Enalapril	89	64	≤0.40	12	–	–	–	–	–	–	–	–	4
HEAVEN [40], 2002	141	Valsartan	Enalapril	75	67	≤0.45	12	–	–	87	–	–	–	–	–	3
Dickstein [39], 1995	166	Losartan	Enalapril	78	64	<0.35	12	–	–	69	3	12	–	23	–	3
ELITE [14], 1997	722	Losartan	Captopril	67	73	≤0.40	48	–	–	68	–	–	25	57	23	4
ELITE II [10], 2000	3152	Losartan	Captopril	70	71	≤0.40	72	–	–	–	–	–	24	49	30	5
RESOLVD [12], 1999	768	Candesartan	Enalapril	84	63	<0.40	43	–	–	72	–	–	–	–	–	5
OPTIMAAL [9], 2002	5477	Losartan	Captopril	71	67	<0.35	130	100	0	0	0	0	17	36	10	5
VALIANT [11], 2003	14,703	Valsartan	Captopril	69	65	≤0.35–0.45	107	100	0	0	0	0	23	55	–	5
Lang [41], 1997	116	Losartan	Enalapril	78	58	≤0.45	12	–	4	47	44	3	–	–	–	3
ACEIs vs Controls																
AIRE [2], 1993	1986	Ramipril	Placebo	74	65	–	60	–	–	–	–	–	12	28	–	5
Balpitt [23], 1998	169	Captopril	Placebo	–	–	–	24	–	–	–	–	–	–	–	–	2
CASSIS [24], 1995	96	Enalapril	Placebo	83	58	<0.40	12	–	–	70	30	–	23	–	–	3
Chalmers [25], 1987	130	Lisinopril	Placebo	69	58	–	12	–	13	48	30	8	–	–	–	2
Colfer [26], 1992	172	Benazepril	Placebo	–	–	≤0.35	12	–	–	–	–	–	–	–	–	2
CONSENSUS [3], 1987	253	Enalapril	Placebo	70	71	–	27	–	–	73	15	26	23	25	58	3
FEST [27], 1995	308	Fosinopril	Placebo	74	63	≤0.35	12	–	–	–	–	–	–	–	–	4
FHFSG [28], 1995	241	Fosinopril	Placebo	80	62	≤0.35	24	–	–	–	–	–	–	–	–	3
Lechat [29], 1993	125	Perindopril	Placebo	–	–	–	12	–	–	–	–	–	–	–	–	3
Newman [30], 1988	105	Captopril	Placebo	–	–	–	12	–	–	–	–	–	–	–	–	2
SAVE [4], 1992	2231	Captopril	Placebo	82	59	≤0.40	144	100	–	–	–	–	21	43	–	5
SOLVD [5], 1991	2569	Enalapril	Placebo	80	61	≤0.35	166	–	–	–	–	–	26	42	10	5
TRACE [6], 1995	1749	Trandolapril	Placebo	72	68	≤0.35	96–200	100	–	–	–	–	14	23	–	5
Aguilar [31], 1999	345	Captopril	Digoxin	68	63	–	216	–	–	–	–	–	–	–	–	3
CARMEN [32], 2008	381	Enalapril	Carvedilol	80	62	<0.40	72	–	–	–	–	–	14	32	17	4
CIBIS III [33], 2011	217	Enalapril	Bisoprolol	71	73	≤0.35	96	–	25	61	12	12	21	59	54	5
Cowley [34], 1994	209	Captopril	Flosequinan	–	–	–	48	–	–	–	–	–	–	–	–	3
Dohmen [35], 1997	266	Captopril	Ibopamine	84	62	<0.40	24	–	6	67	27	–	8	–	15	3
Hy-C [36], 1992	104	Captopril	Hydralazine	86	52	0.2(m)	32	–	–	59	34	4	–	–	17	3
IMPRESS [37], 2007	573	Lisinopril	Omapatrilat	89	64	–	>40	–	4	66	24	3	–	–	–	5
Northridge [38], 1999	45	Captopril	Candoxatril	87	63	<0.40	12	–	–	–	–	–	–	–	–	2
OVERTURE [7], 2002	5770	Enalapril	Omapatrilat	79	63	≤0.30	58	–	–	56	–	–	31	–	–	5
V-HeFT II [8], 1991	804	Enalapril	Nitrates	–	61	<0.45	96	–	–	–	–	–	20	48	14	3

Data was absent in the original article

*No* number, *LVEF* left ventricular ejection fraction, *MI* myocardial infarction, *HTN* hypertension, *DM* diabetes mellitus, *AF* atrial fibrillation, *ACEI* angiotensin-converting enzyme inhibitors, *ARB* angiotensin II Receptor Blockers, *ICM* ischemic cardiomyopathy, *NICM* non-ischemic cardiomyopathy, *VHD* valvular heart disease, *m* mean

### Effect of ACEIs and ARBs on all-cause mortality

**Fig. 2:**
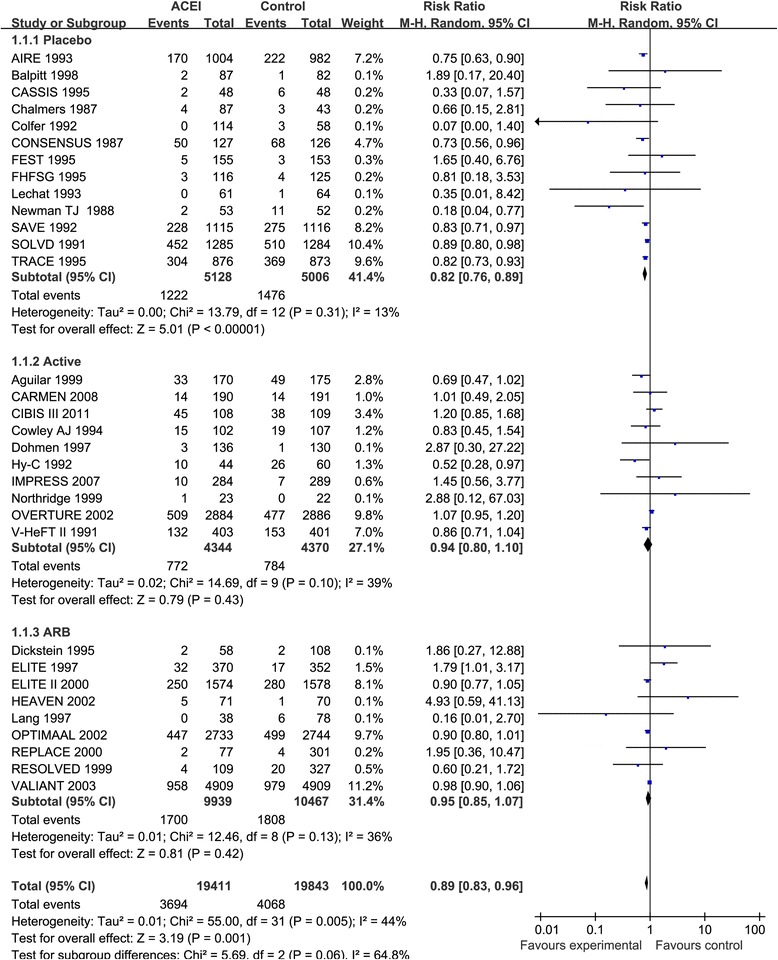
Forest plot of angiotensin-converting enzyme inhibitors (ACEIs) compared with controls on all-cause mortality. Boxes and solid lines indicate RR and 95%CI, respectively for each study, and the diamonds and their width indicate the pooled RR and the 95% CI, respectively. M-H indicates Mantel-Haenszel. ACEI, angiotensin-converting enzyme inhibitor, ARB, angiotensin II receptor blocker

Thirty-two studies [2–12, 14, 23–42] reported the effect of ACEIs on all-cause mortality in a total of 39,254 HF patients with moderate heterogeneity in overall analysis (I^2^ = 44%, *p* = 0.005). ACEIs were associated with a statistically significant 11% reduction in all-cause mortality (RR: 0.89, 95% CI: 0.83–0.96, *p* = 0.001, Fig. 2). Similar findings were observed when ACEIs were compared with placebo treatment (*p* < 0.001, Fig. 2). There was no evidence of publication bias (*p* = 0.833).

**Fig. 3:**
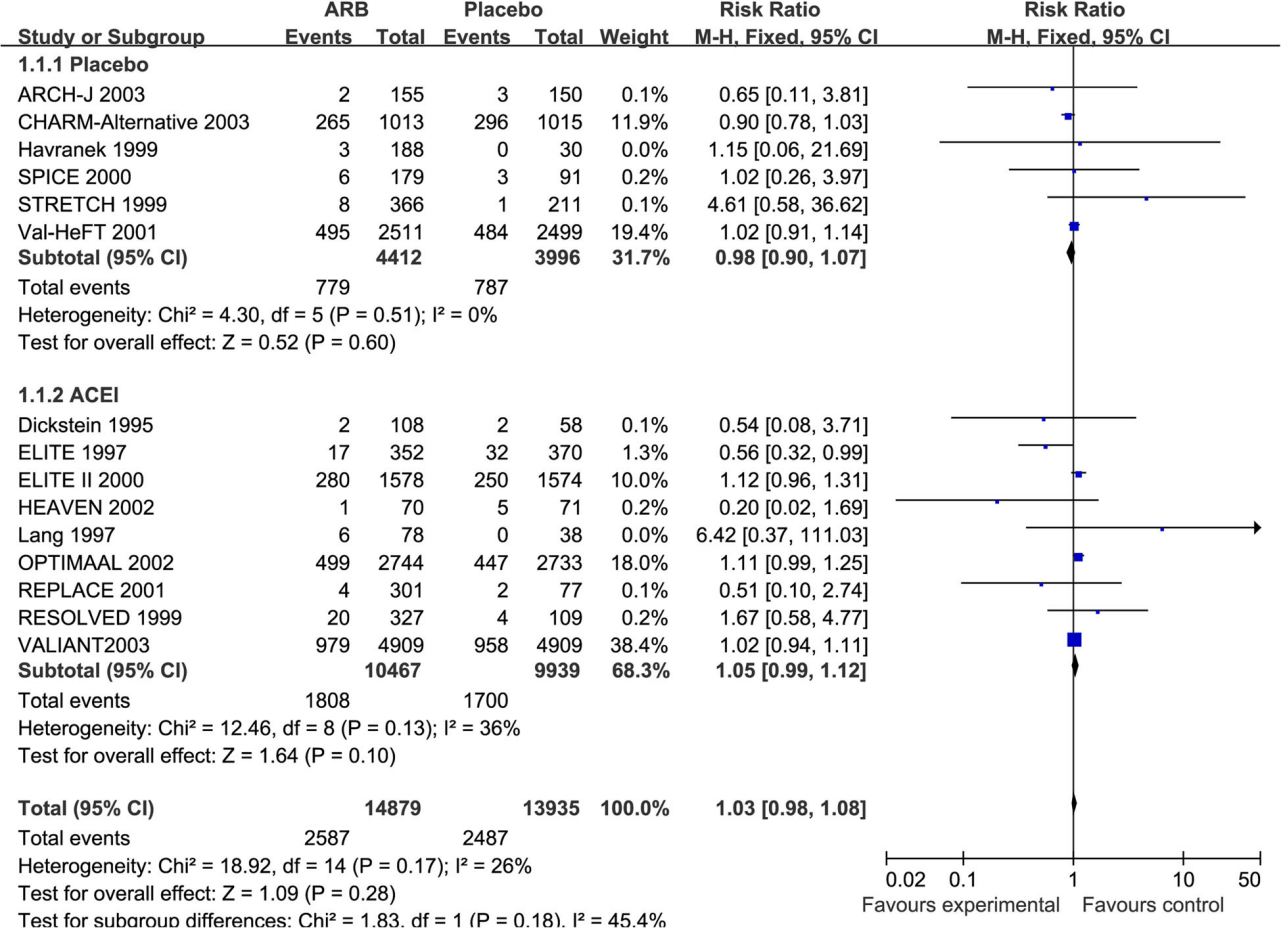
Forest plot of angiotensin II receptor blocker inhibitors (ARBs) compared with controls on all-cause mortality. Boxes and solid lines indicate RR and 95%CI, respectively for each study, and the diamonds and their width indicate the pooled RR and the 95% CI, respectively. M-H indicates Mantel-Haenszel. ACEI, angiotensin-converting enzyme inhibitor, ARB, angiotensin II receptor blocker

Moreover, 15 studies [9–14, 39–47] reported the effect of ARBs on all-cause mortality in a total of 28,814 HF patients with no significant heterogeneity in overall analysis (I^2^ = 26%, *p* = 0.17). ARBs were not associated with a reduction in all-cause mortality (RR: 1.03, 95% CI: 0.98–1.08, *p* = 0.28, Fig. 3). Similar findings were observed when comparing with placebo or ACEIs (*p* ≤ 0.60, Fig. 3). And there was no evidence of publication bias (*p* = 0.921).

**Fig. 4:**
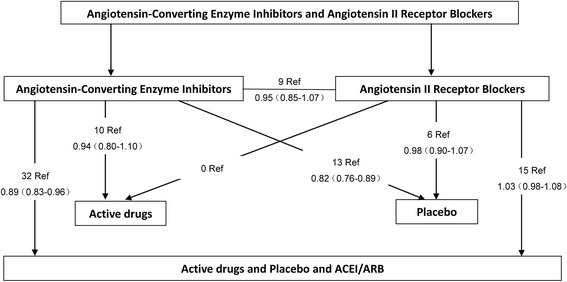
Randomised controlled trials comparing effect of ACEIs and ARB treatment on all-cause mortality. Summary risk ratios (95%confidence intervals) are shown for each comparison. ACEI, angiotensin-converting enzyme inhibitor, ARB, angiotensin II receptor blocker

Figure 4 showed the relation between the network of RCTs.

### Effect of ACEIs and ARBs on CV mortality

**Fig. 5:**
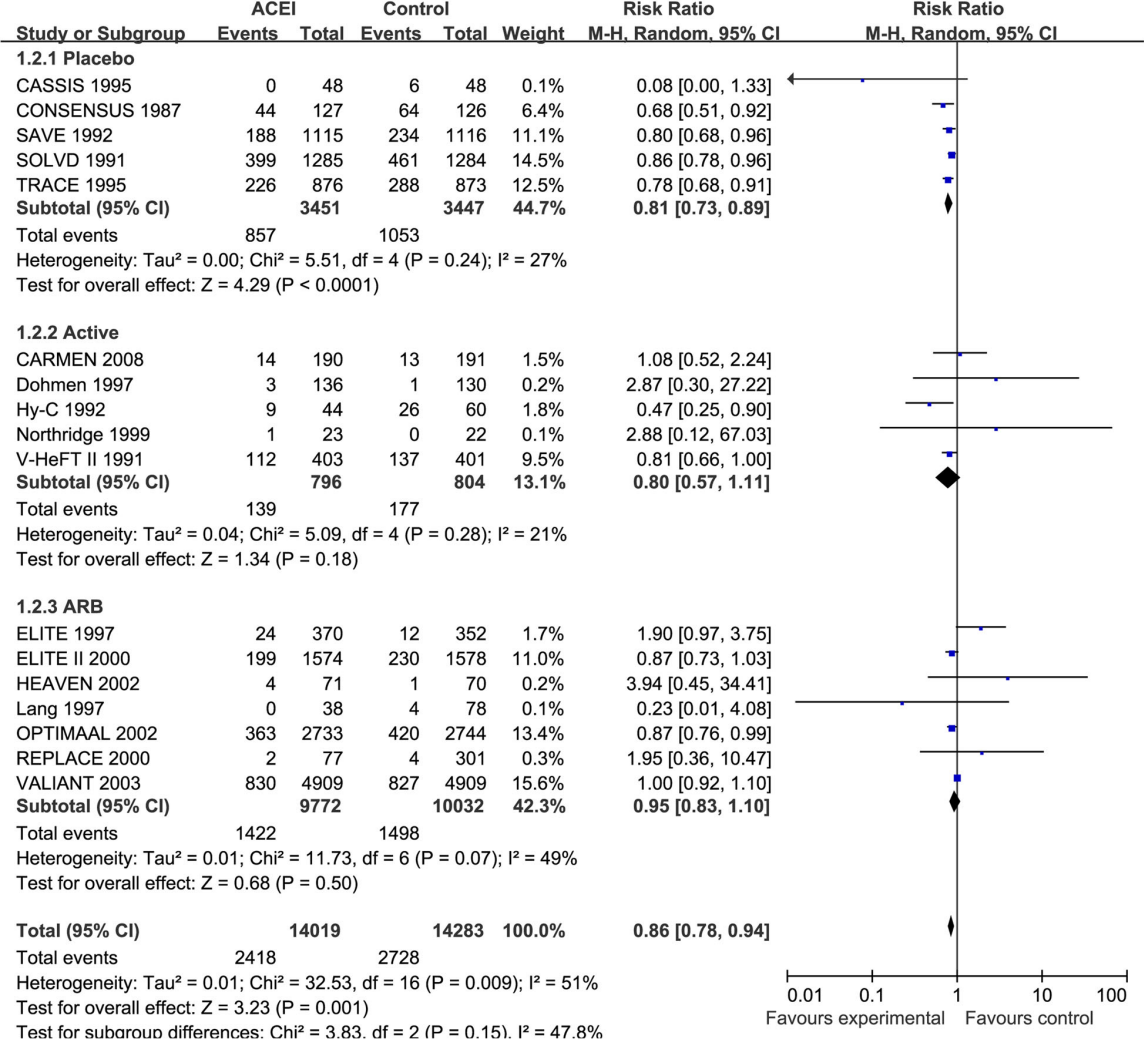
Forest plot of angiotensin-converting enzyme inhibitors (ACEIs) compared with controls on cardiovascular mortality. Boxes and solid lines indicate RR and 95%CI, respectively for each study, and the diamonds and their width indicate the pooled RR and the 95% CI, respectively. M-H indicates Mantel-Haenszel. ACEI, angiotensin-converting enzyme inhibitor, ARB, angiotensin II receptor blocker

Seventeen studies [3–6, 8–11, 14, 24, 32, 35, 36, 38, 40–42] reported the effectiveness of ACEIs for CV mortality in a total of 28,302 HF patients with moderate heterogeneity in overall analysis (I^2^ = 51%, *p* = 0.009). ACEIs were associated with a statistically significant 14% reduction in CV mortality (RR: 0.86, 95% CI: 0.78–0.94, *p* = 0.001, Fig. 5). Similar findings were observed when ACEIs treatment was compared with placebo treatment (*p* < 0.001, Fig. 5). However, when ACEIs were compared with active treatment or ARBs, ACEIs did not significantly reduce CV mortality. There was no evidence of publication bias (*p* = 0.967). The SAVE [4], TRACE [6] and VALIANT [11] study were conducted in patients with HF after myocardial infarction. After exclusion of these three trials, heterogeneity among the trials was not significantly different (I^2^ = 34%, *p* = 0.10, RR, 0.85, 95% CI: 0.76–0.95, *p* = 0.005).

Moreover, 11 studies [9–11, 13, 14, 40–42, 45–47] reported the effectiveness of ARBs for CV mortality in a total of 27,991 HF patients with no significant heterogeneity in overall analysis (I^2^ = 40%, *p* = 0.08). ARBs were associated with no reduction in CV mortality (RR: 1.01, 95% CI: 0.92–1.12, *p* = 0.78, Additional file 1: Figure S1). Similar findings were observed when ARBs were compared with placebo or ACEIs (*p* ≤ 0.50, Additional file 1: Figure S1). And there was no evidence of publication bias (*p* = 1.000).

### Meta-regression

**Fig. 6:**
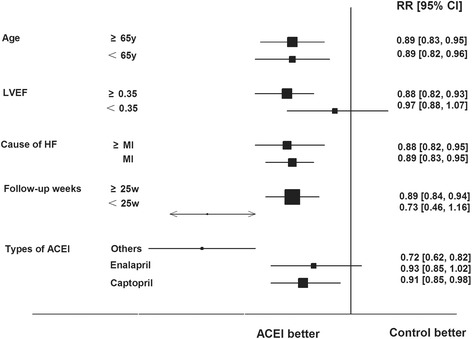
Univariate Meta-regression Analysis of Potential Sources of Heterogeneity on Effect of ACEIs on All-Cause Mortality. Boxes and solid lines indicate RR and 95%CI, respectively for each study, and the diamonds and their width indicate the pooled RR and the 95% CI, respectively. Trials to the left of the vertical line showed a reduction in risk with the experimental intervention; those to the right showed an increase in risk with the experimental intervention. ACEI, angiotensin-converting enzyme inhibitor, MI, myocardial infraction, HF, heart failure, LVEF, left ventricular ejection fraction

Meta-regression was conducted in different ages (*p* = 0.97), causes of HF (*p* = 0.90), left ventricular ejection fractions (*p* = 0.09), follow-up weeks (*p* = 0.41) to observe effects of ACEIs treatment on all-cause mortality. The findings remained unaltered in these subgroup analyses. But, univariate meta-regression of ACEIs treatment on all-cause mortality varied by the types of ACEIs (*p* = 0.004). Captopril treatment reduced all-cause mortality by 9% (RR: 0.91, 95% CI: 0.85–0.98, *p* = 0.008) in HF patients as compared with control treatment. However, enalapril treatment did not reduce all-cause mortality in HF patients as compared with control treatment (RR: 0.93, 95% CI: 0.85–1.02, *p* = 0.13, Fig. 6). Of all these studies, one study compared ramipril with placebo and two studies compared lisinopril with placebo/active drugs. The results were shown in Fig. 5.

## Discussion

### Major findings

In this meta-analysis, we combined clinical trial data from 38 studies, which included 47,662 HF patients to assess the efficacy of RAAS inhibition on mortality. Overall, ACEIs reduce all-cause mortality by 11% and the corresponding value for CV mortality by 14%. However, ARBs have no significant effect on all-cause and CV mortality in HF patients. In head-to-head analysis, ACEIs are not superior to ARBs on all-cause and CV mortality. Thus, this meta-analysis provides compelling evidence that ACEIs are the most effective first-line treatment for preventing all-cause and CV mortality in HF patients.

RAAS inhibition has long been identified as a preferred first-line treatment for heart failure. However, previous studies indicated that there were different outcomes between AECIs and ARBs for heart failure. As early as 1987, CONSENSUS study [3] was conducted to evaluate the efficiency of enalapril in patients with HF. Six-month mortality in the enalapril group was 26% compared with 44% in the placebo group, giving a relative risk-reduction of 40% (*p* = 0.002) and at 1 year, these proportions were 36% and 52% (*p* = 0.001). After that, several studies demonstrated that ACEIs reduced all-cause and CV mortality in HF patients, particularly after myocardial infarction [4, 6]. However, most of ARBs are not proved to be effective on these crucial outcomes in HF patients. For example, the ELITE II study [10] found that losartan is not superior to captopril, although it has been suggested that the dose of losartan (50 mg) tested is not adequate. And in the CHARM-Alternative trial [13], candesartan did not reduce all-cause mortality in HF patients, but reduced the risk of CV death or HF hospitalization by 23% (*p* = 0.0004). The Val-Heft study46 showed the same results. These may due to the negative effect of ARBs on heart failure, which could be mediated through a vasoconstrictor-induced increase in blood pressure or a direct effect on cardiac and vascular tissues. So, more related studies are expected to conducted in this area. Besides, in some recent meta-analysis, Vark et al. [15] and Cheng et al. [16] presented that in patients with hypertension and diabetes, treatment with an ACEI resulted in a significant further reduction in all-cause and CV mortality, whereas ARBs had no benefit on these outcomes. These results are in agreement with our meta-analysis.

### Pharmacological mechanism

From a pharmacological viewpoint, ACEIs can reduce the negative effects caused by binding of angiotensin II and its receptor by inhibiting the conversion of angiotensin I to angiotensin II. In addition, by restraining the degradation of angiotensin (1–7) and promoting its combination with Mas receptor, ACEIs may have effect on dilating blood vessels, anti-inflammatory and anti-fibrosis. Moreover, ACEIs can also reduce degradation of bradykinin and promote its role in β2 receptor, which contributes to dilation of blood vessels, anti-proliferation, endothelial protection and other positive effects [48, 49]. In contrast, RAAS blockade with ARBs is achieved by inhibiting the binding of angiotensin II to the angiotensin II type one receptor, which is believed to mediate the harmful cardiovascular effects of angiotensin II due to the elevated level of angiotensin II by compensatory mechanism. These different pharmacological mechanisms may explain that ACEIs may be superior to ARBs in reducing CV events. Therefore, an ACEI agent may be a superior ARB antagonist in hypertension and heart failure.

### Heterogeneity

There was low to moderate heterogeneity of analysis on the effect of ACEIs on all-cause and CV mortality. Meta-regression, sensitivity and subgroup analysis were conducted to estimate the influence of each study. Firstly, no evidence shows that the observed effects varied by age, causes of HF, left ventricular ejection fractions and follow-up weeks by meta-regression. However, different types of ACEIs may influence the effect on all-cause mortality, which means that captopril may be superior to enalapril in reducing all-cause mortality in HF patients. Secondly, the SAVE [4], TRACE [6] and VALIANT [11] study were conducted in patients with heart failure after myocardial infarction. After exclusion of these three trials, heterogeneity among the trials exploring the effect of ACEIs on CV mortality was not significantly different (I^2^ = 34%, *p* = 0.10, RR, 0.85, 95% CI: 0.76–0.95, *p* = 0.005). So, the significant heterogeneity was attributable to the different control treatment. There was no evidence of publication bias (*p* = 0.721).

### Study strengths and limitations

Strengths of the present study are no other than the large sample size with a mean/median follow-up ranging from 12 weeks to 4.5 years and a high representativeness.

It has been acknowledged that there are some limitations to this study. Firstly, this analysis used aggregate data as reported or calculated in published articles, rather than data of individual patients. Secondly, there were a great deal of variations between the studied populations. For example, causes of heart failure differed from each other. In addition, these trials used different ACEIs or ARBs at a different dosage. It is likely that different ACEIs and ARBs may have a total different effect on the cardiac mortality. Moreover, the present study is unable to address whether the efficacy may be varied in HF patients with different ethnic backgrounds.

## Conclusions

In 47,662 subjects, our meta-analysis shows that ACEIs, but not ARBs reduce all-cause mortality and cardiovascular deaths in HF patients. Thus, ACEIs should be considered as first-line therapy to limit excess mortality and morbidity in this population.

## Additional file

Additional file 1: Figure S1. Forest plot of angiotensin II receptor blocker inhibitors (ARBs) compared with controls on cardiovascular mortality. Boxes and solid lines indicate RR and 95%CI, respectively for each study, and the diamonds and their width indicate the pooled RR and the 95% CI, respectively. Trials to the left of the vertical line showed a reduction in risk with the experimental intervention; those to the right showed an increase in risk with the experimental intervention. M-H indicates Mantel-Haenszel. ACEI, angiotensin-converting enzyme inhibitor, ARB, angiotensin II receptor blocker. (TIFF 785 kb)

## Abbreviations

ACEIs: Angiotensin-converting enzyme inhibitors
ARBs: Angiotensin II receptor blockers
CI: Confidence interval
CV: Cardiovascular
HF: Heart failure
LVEF: Left ventricular ejection fraction
RAAS: Rennin angiotensin aldosterone system
RCT: Randomized clinical trial
RR: Risk ratio

## References

[1] McMurray JJ, Adamopoulos S, Anker SD, Auricchio A, Böhm M, Dickstein K (2012). ESC guidelines for the diagnosis and treatment of acute and chronic heart failure 2012. Eur J Heart Fail.

[2] The Acute Infarction Ramipril Efficacy (AIRE) Study Group (1993). Effect of ramipril on mortality and morbidity of survivors of acute myocardial infarction with clinical evidence of heart failure. Lancet.

[3] CONSENSUS Trial Study Group (1987). Effects of enalapril on mortality in severe congestive heart failure. Results of the Cooperative North Scandinavian Enalapril Survival Study (CONSENSUS). N Engl J Med.

[4] Pfeffer M, Braunwald E, Moyé L, Basta L, Brown E, Cuddy T (1992). Effect of captopril on mortality and morbidity in patients with left ventricular dysfunction after myocardial infarction: results of the survival and ventricular enlargement trial. N Engl J Med.

[5] Investigators S (1991). Effect of enalapril on survival in patients with reduced left ventricular ejection fraction and congestive heart failure. N Engl J Med.

[6] Køber L, Torp-Pedersen C, Carlsen JE, Bagger H, Eliasen P, Lyngborg K (1995). A clinical trial of the angiotensin-converting–enzyme inhibitor trandolapril in patients with left ventricular dysfunction after myocardial infarction. N Engl J Med.

[7] Packer M, Califf RM, Konstam MA, Krum H, McMurray JJ, Rouleau JL (2002). Comparison of omapatrilat and enalapril in patients with chronic heart failure the Omapatrilat Versus Enalapril Randomized Trial of Utility in Reducing Events (OVERTURE). Circulation.

[8] Cohn JN, Johnson G, Ziesche S, Cobb F, Francis G, Tristani F (1991). A comparison of enalapril with hydralazine–isosorbide dinitrate in the treatment of chronic congestive heart failure. N Engl J Med.

[9] Dickstein K, Kjekshus J (2002). Effects of losartan and captopril on mortality and morbidity in high-risk patients after acute myocardial infarction: the OPTIMAAL randomised trial. Lancet.

[10] Pitt B, Poole-Wilson PA, Segal R, Martinez FA, Dickstein K, Camm AJ (2000). Effect of losartan compared with captopril on mortality in patients with symptomatic heart failure: randomised trial—the Losartan Heart Failure Survival Study ELITE II. Lancet.

[11] Pfeffer MA, McMurray JJV, Velazquez EJ, Rouleau JL, Køber L, Maggioni AP (2003). Valsartan, captopril, or both in myocardial infarction complicated by heart failure, left ventricular dysfunction, or both. N Engl J Med.

[12] McKelvie RS, Yusuf S, Pericak D, Avezum A, Burns RJ, Probstfield J (1999). Comparison of candesartan, enalapril, and their combination in congestive heart failure Randomized Evaluation of Strategies for Left Ventricular Dysfunction (RESOLVD) pilot study: the RESOLVD pilot study investigators. Circulation.

[13] Granger CB, McMurray JJV, Yusuf S, Held P, Michelson EL, Olofsson B (2003). Effects of candesartan in patients with chronic heart failure and reduced left-ventricular systolic function intolerant to angiotensin-converting-enzyme inhibitors: the CHARM-alternative trial. Lancet.

[14] Pitt B, Segal R, Martinez FA, Meurers G, Cowley AJ, Thomas I (1997). Randomised trial of losartan versus captopril in patients over 65 with heart failure (Evaluation of Losartan in the Elderly Study, ELITE). Lancet.

[15] van Vark LC, Bertrand M, Akkerhuis KM, Meurers G, Cowley AJ, Thomas I (2012). Angiotensin-converting enzyme inhibitors reduce mortality in hypertension: a meta-analysis of randomized clinical trials of renin–angiotensin–aldosterone system inhibitors involving 158 998 patients. Eur Heart J.

[16] Cheng J, Zhang W, Zhang X, Han F, Li X, He X (2014). Effect of angiotensin-converting enzyme inhibitors and angiotensin ii receptor blockers on all-cause mortality, cardiovascular deaths, and cardiovascular events in patients with diabetes mellitus: a meta-analysis. JAMA Inter Med.

[17] Moher D, Liberati A, Tetzlaff J, Altman DG (2009). Preferred reporting items for systematic reviews and meta-analyses: the PRISMA statement. Ann Intern Med.

[18] Jadad AR, Moore RA, Carroll D, Jenkinson C, Reynolds DJ, Gavaghan DJ (1996). Assessing the quality of reports of randomized clinical trials: is blinding necessary?. Control Clin Trials.

[19] Ades AE, Sculpher M, Sutton A, Abrams K, Cooper N, Welton N (2006). Bayesian methods for evidence synthesis in cost-effectiveness analysis. PharmacoEconomics.

[20] Higgins JP, Whitehead A (1996). Borrowing strength from external trials in a meta-analysis. Stat Med.

[21] Caldwell DM, Ades AE, Higgins JP (2005). Simultaneous comparison of multiple treatments: combining direct and indirect evidence. BMJ.

[22] Higgins J, Green S. Cochrane handbook for systematic reviews of interventions Version 5.1.0 [updated March 2011]. The Cochrane Collaboration, 2011. Available from http://training.cochrane.org/handbook.

[23] Bulpitt CJ, Fletcher AE, Dössegger L, Neiss A, Nielsen T, Viergutz S (1998). Quality of life in chronic heart failure: cilazapril and captopril versus placebo. Heart.

[24] Widimský J, Uhlíř O, Kremer HJ, Jerie P (1995). Czech and Slovak spirapril intervention study (CASSIS). Eur J Clin Pharmacol.

[25] Chalmers JP, West MJ, Cyran J, De La Torre D, Englert M, Kramar M (1987). Placebo-controlled study of lisinopril in congestive heart failure: a multicentre study. J Cardiovasc Pharmacol.

[26] Colfer HT, Ribner HS, Gradman A, Hughes CV, Kapoor A, Laidlaw JC (1992). Effects of once-daily benazepril therapy on exercise tolerance and manifestations of chronic congestive heart failure. Am J Cardiol.

[27] Erhardt L, MacLean A, Ilgenfritz J, Gelperin K, Blumenthal M (1995). Fosinopril attenuates clinical deterioration and improves exercise tolerance in patients with heart failure. Eur Heart J.

[28] Brown EJ, Chew PH, MacLean A, Gelperin K, Ilgenfritz JP (1995). BlumenthalM. Effects of fosinopril on exercise tolerance and clinical deterioration in patients with chronic congestive heart failure not taking digitalis. Am J Cardiol.

[29] Lechat P, Garnham SP, Desche P, Bounhoure JP (1993). Efficacy and acceptability of perindopril in mild to moderate chronic congestive heart failure. Am Heart J.

[30] Newman TJ, Maskin CS, Dennick LG, Meyer JH, Hallows BG, Cooper WH (1988). Effects of captopril on survival in patients with heart failure. Am J Med.

[31] Cosin-Aguilar J, Marrugat J, Sanz G, Massó J, Gil M, Vargas R (1999). Long-term results of the Spanish trial on treatment and survival of patients with predominantly mild heart failure. J Cardiovasc Pharmacol.

[32] Komajda M, Lutiger B, Madeira H, Thygesen K, Bobbio M, Hildebrandt P (2004). Tolerability of carvedilol and ACE-inhibition in mild heart failure. Results of CARMEN (Carvedilol ACE-Inhibitor Remodelling Mild CHF EvaluatioN). Eur J Heart Fail.

[33] Funck-Brentano C, Veldhuisen DJ, Ven LLM, Follath F, Goulder M, Willenheimer R (2011). Influence of order and type of drug (bisoprolol vs enalapril) on outcome and adverse events in patients with chronic heart failure: a post hoc analysis of the CIBIS-III trial. Eur J Heart Fail.

[34] Cowley AJ, McEntegart DJ, Hampton JR, Barnett DB, Bexton RS, Boyle R (1994). Long-term evaluation of treatment for chronic heart failure: a 1 year comparative trial of flosequinan and captopril. Cardiovasc Drug Ther.

[35] Dohmen HJ, Dunselman PH, Poole-Wilson PA (1997). Comparison of captopril and ibopamine in mild to moderate heart failure. Heart.

[36] Fonarow GC, Chelimsky-Fallick C, Stevenson LW, Luu M, Hamilton MA, Moriguchi JD (1992). Effect of direct vasodilation with hydralazine versus angiotensin-converting enzyme inhibition with captopril on mortality in advanced heart failure: the Hy-C trial. J Am Coll Cardiol.

[37] Rouleau JL, Pfeffer MA, Stewart DJ, Isaac D, Sestier F, Kerut EK (2000). Comparison of vasopeptidase inhibitor, omapatrilat, and lisinopril on exercise tolerance and morbidity in patients with heart failure: IMPRESS randomised trial. Lancet.

[38] Northridge DB, Currie PF, Newby DE, McMurray JJ, Ford M, Boon NA, Dargie HJ (1999). Placebo-controlled comparison of candoxatril, an orally active neutral endopeptidase inhibitor, and captopril in patients with chronic heart failure. Eur J Heart Fail.

[39] Dickstein K, Chang P, Willenheimer R, Haunsø S, Remes J, Hall C (1995). Comparison of the effects of losartan and enalapril on clinical status and exercise performance in patients with moderate or severe chronic heart failure. J Am Coll Cardiol.

[40] Willenheimer R, Helmers C, Pantev E, Rydberg E, Löfdahl P, Gordon A (2002). Safety and efficacy of valsartan versus enalapril in heart failure patients. Int J Cardiol.

[41] Lang RM, Elkayam U, Yellen LG, Krauss D, McKelvie RS, Vaughan DE (1997). Comparative effects of losartan and enalapril on exercise capacity and clinical status in patients with heart failure. J Am Coll Cardiol.

[42] Dunselman P (2001). Effects of the replacement of the angiotensin converting enzyme inhibitor enalapril by the angiotensin II receptor blocker telmisartan in patients with congestive heart failure: the replacement of angiotensin converting enzyme inhibition (REPLACE) investigators. Int J Cardiol.

[43] Havranek EP, Thomas I, Smith WB, Ponce GA, Bilsker M, Munger MA (1999). Dose-related beneficial long-term hemodynamic and clinical efficacy of irbesartan in heart failure. J Am Coll Cardiol.

[44] Granger CB, Ertl G, Kuch J, Maggioni AP, McMurray J, Rouleau JL (2000). Randomized trial of candesartan cilexetil in the treatment of patients with congestive heart failure and a history of intolerance to angiotensin-converting enzyme inhibitors. Am Heart J.

[45] Riegger GAJ, Bouzo H, Petr P, Münz J, Spacek R, Pethig H (1999). Improvement in exercise tolerance and symptoms of congestive heart failure during treatment with candesartan cilexetil. Circulation.

[46] Cohn JN, Tognoni G (2001). A randomized trial of the angiotensin-receptor blocker valsartan in chronic heart failure. N Engl J Med.

[47] Matsumori A (2003). Efficacy and safety of oral candesartan cilexetil in patients with congestive heart failure. Eur J Heart Fail.

[48] Goussev A, Sharov VG, Shimoyama H, Tanimura M, Lesch M, Goldstein S (1998). Effects of ACE inhibition on cardiomyocyte apoptosis in dogs with heart failure. Am J Physiol.

[49] Pahor M, Bernabei R, Sgadari A, Gambassi G, Lo Giudice P, Pacifici L (1991). Enalapril prevents cardiac fibrosis and arrhythmias in hypertensive rats. Hypertension.

