# Supplementary figures and images for: Effect of angiotensin-converting enzyme inhibitors and angiotensin II receptor blockers on cardiovascular events in patients with heart failure: a meta-analysis of randomized controlled trials
Source: BMC Cardiovasc Disord. 2017 Oct 5;17:257. doi: 10.1186/s12872-017-0686-z (PMC5629775; doi:10.1186/s12872-017-0686-z)

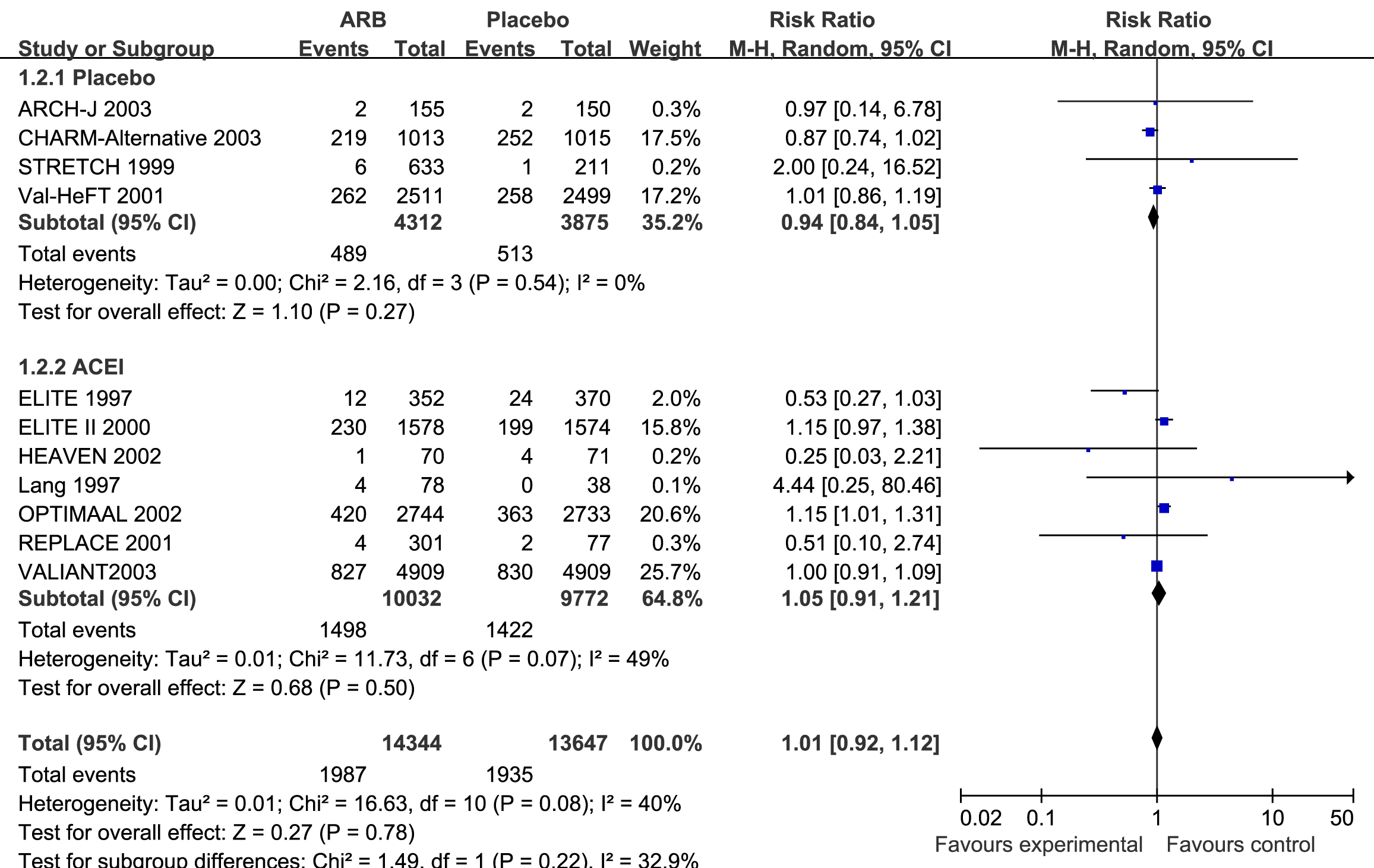

Supplement: Additional file 1: Figure S1. — Forest plot of angiotensin II receptor blocker inhibitors (ARBs) compared with controls on cardiovascular mortality. Boxes and solid lines indicate RR and 95%CI, respectively for each study, and the diamonds and their width indicate the pooled RR and the 95% CI, respectively. Trials to the left of the vertical line showed a reduction in risk with the experimental intervention; those to the right showed an increase in risk with the experimental intervention. M-H indicates Mantel-Haenszel. ACEI, angiotensin-converting enzyme inhibitor, ARB, angiotensin II receptor blocker. (TIFF 785 kb) [file 12872_2017_686_MOESM1_ESM.tif]
